# Supplementary material for: Mutations associated with human neural tube defects display disrupted planar cell polarity in Drosophila
Source: eLife. 2020 Apr 1;9:e53532. doi: 10.7554/eLife.53532 (PMC7180057; doi:10.7554/eLife.53532)
Supplement: Figure 1—source data 1. [file elife-53532-fig1-data1.docx]

| *mVL1* | *mVL2* | *Mutation* | *Drosophila* | *Conserved* | *Dvl binding* | *PolyPhen2* | *SIFT* | *References* |
| --- | --- | --- | --- | --- | --- | --- | --- | --- |
|  | *D255E* | *Conservative* | *D317* | *Absolutely* | *No* | *PrD* | *D* | *Kibar et al. 2001* |
|  | *R259L* | *Basic-Aliphatic* | *R321* | *Highly* | *ND* | *PrD* | *D* | *Guyot et al. 2011* |
|  | *I268N* | *Aliphatic-Polar* | *I330* | *Absolutely* | *No* | *PrD* | *D* | *El-Hassan et al. 2017* |
|  | *Q449$* | *Protein truncation* | *Q512* | *N/A* | *ND* | *N/A* | *N/A* | *Chen et al. 2013* |
|  | *S464N* | *Conservative* | *C527* | *Highly* | *No* | *PrD* | *D* | *Kibar et al. 2001* |

| *hVL1* | *hVL2* | *Mutation* | *Drosophila* | *Conserved* | *Dvl binding* | *PolyPhen2* | *SIFT* | *References* |
| --- | --- | --- | --- | --- | --- | --- | --- | --- |
|  | *L242V* | *Conservative* | *Q304* | *Highly** | *ND* | *PrD* | *D* | *Kibar et al. 2011* |
| *T251M* | *T247M* | *Polar-Hydrophobic* | *Y309* | *Moderately* | *ND* | *PrD* | *T* | *Kibar et al. 2009 & 2011* |
| *R274Q* | *R270H* | *Basic-Polar / Conservative* | *R332* | *Absolutely* | *Yes* | *PrD* | *D* | *Kibar et al. 2007 & 2011* |
| *Y290H* |  | *Aromatic-Basic* | *F348* | *Moderately* | *ND* | *Benign* | *T* | *Kibar et al. 2009* |
| *M328T* |  | *Hydrophobic-Polar* | *V391* | *Moderately* | *Yes* | *Benign* | *T* | *Kibar et al. 2007* |
|  | *R353C* | *Basic-Polar* | *K418* | *Highly* | *Yes*** | *PrD* | *D* | *Lei et al. 2010* |
| *D389H* |  | *Acidic-Basic* | *D449* | *Highly* | *ND* | *PrD* | *D* | *Merello et al. 2015* |
| *A404S* |  | *Hydrophobic-Polar* | *A464* | *Highly* | *ND* | *Benign* | *T* | *Kibar et al. 2009* |
|  | *F437S* | *Aromatic-Polar* | *F500* | *Absolutely* | *No* | *PrD* | *D* | *Lei et al. 2010* |
| *D468E**** |  | *Conservative* | *D528* | *Highly* | *ND* | *Benign* | *T* | *Kibar et al. 2009* |
|  | *R482H* | *Conservative* | *Q545* | *Moderately* | *ND* | *Benign* | *T* | *Kibar et al. 2011* |
| *R517H* |  | *Conservative* | *K577* | *Highly* | *ND* | *PrD* | *T* | *Merello et al. 2015* |

*ND – Not Determined; PrD – Probably Damaging; D – Damaging; T – Tolerated; * not conserved in Drosophila;*

***a potential reduction in binding was reported; *** polymorphism*
